# First genetic insights of *Gonatodescaudiscutatus* (Reptilia, Gekkota) in the Galapagos Islands and mainland Ecuador

**DOI:** 10.3897/BDJ.11.e113396

**Published:** 2023-11-16

**Authors:** Lía Altamirano-Ponce, Mateo Dávila-Játiva, Gabriela Pozo, María José Pozo, Martín Terán-Velástegui, Carlos Daniel Cadena, Diego F. Cisneros-Heredia, Maria de Lourdes Torres

**Affiliations:** 1 Universidad San Francisco de Quito USFQ, Colegio de Ciencias Biológicas y Ambientales, Instituto de Biodiversidad Tropical IBIOTROP, Laboratorio de Zoología Terrestre, Quito, Ecuador Universidad San Francisco de Quito USFQ, Colegio de Ciencias Biológicas y Ambientales, Instituto de Biodiversidad Tropical IBIOTROP, Laboratorio de Zoología Terrestre Quito Ecuador; 2 Universidad San Francisco de Quito USFQ, Colegio de Ciencias Biológicas y Ambientales, Laboratorio de Biotecnología Vegetal, Quito, Ecuador Universidad San Francisco de Quito USFQ, Colegio de Ciencias Biológicas y Ambientales, Laboratorio de Biotecnología Vegetal Quito Ecuador; 3 Universidad San Francisco de Quito USFQ, extensión Galápagos GAIAS, Puerto Baquerizo Moreno, San Cristóbal, Galápagos, Ecuador Universidad San Francisco de Quito USFQ, extensión Galápagos GAIAS, Puerto Baquerizo Moreno, San Cristóbal Galápagos Ecuador; 4 Universidad de los Andes, Departamento de Ciencias Biológicas, Laboratorio de Biología Evolutiva de Vertebrados, Bogotá, Colombia Universidad de los Andes, Departamento de Ciencias Biológicas, Laboratorio de Biología Evolutiva de Vertebrados Bogotá Colombia; 5 Galápagos Science Center, Universidad San Francisco de Quito USFQ & University of North Carolina at Chapel Hill UNC, Galápagos, Ecuador Galápagos Science Center, Universidad San Francisco de Quito USFQ & University of North Carolina at Chapel Hill UNC Galápagos Ecuador

**Keywords:** introduced species, genetic variability, haplotype, gecko, *Gonatodescaudiscutatus*, Galapagos Islands, Ecuador

## Abstract

Studies on genetic variability amongst native and introduced species contribute to a better understanding of the genetic diversity of species along their autochthonous distribution and identify possible routes of introduction. *Gonatodescaudiscutatus* is a gecko native to western Ecuador and introduced to the Galapagos Islands. Despite being a successful species in human-modified habitats along its native and non-native ranges, neither the colonisation process nor the genetic diversity of this gecko is known. In this study, we analysed 55 individuals from 14 localities in western Ecuador and six localities in San Cristobal Island, Galapagos — the only island with a large, self-sustaining population. We amplified and analysed the genetic variability of two nuclear genes (Cmos and Rag2) and one mitochondrial gene (16S). Cmos and Rag2 sequences presented little to none genetic variability, while 16S allowed us to build a haplotype network. We identified nine haplotypes across mainland Ecuador, two of which are also present in Galapagos. Low genetic diversity between insular and continental populations suggests that the introduction of *G.caudiscutatus* on the Islands is relatively recent. Due to the widespread geographical distribution of mainland haplotypes, it was not possible to determine the source population of the introduction. This study represents the first exploration of the genetic diversity of *Gonatodescaudiscutatus*, utilising genetic tools to gain insights into its invasion history in the Galapagos.

## Introduction

Human-mediated introductions of non-native species are a common and well-documented phenomenon, exponentially increasing in our globalised world (Kraus 2009a, Seebens et al. 2017). Human-introduced species tend to thrive in insular and mainland coastal ecosystems to the point of displacing their native ecological counterparts (Hoskin 2011, Michaelides et al. 2019). Extensive research has been conducted to explore contemporary biological invasions, identifying the underlying mechanisms driving them and evaluating their impacts (Dame and Petren 2006, Yoshida et al. 2007, Keller and Taylor 2008, Yang et al. 2011). Genetic information from non-native and native populations of invasive species can help identify possible invasion routes and mechanisms and, thus, develop better conservation management tools to mitigate the impact of invasive species (Sakai et al. 2001, Kolbe et al. 2004, Abdelkrim et al. 2005, Frankham 2009, Peh 2009, Lu et al. 2020). Recent investigations of human-mediated introductions of lizards have shown a diversity of dynamic processes involved, including marginal reductions in genetic diversity and shifts in haplotype frequencies due to admixture of genotypes from different source populations, multiple introductions, large numbers of founding individuals, gene flow, natural selection and hybridisation (Kolbe et al. 2004, Kolbe et al. 2008, Schulte et al. 2012, Detwiler and Criscione 2014, Moule et al. 2015).

Geckos (infraorder Gekkota) are some of the most successful colonisers of novel areas amongst terrestrial vertebrate taxa. Many species in this group have established thriving non-native populations on oceanic islands (Lever 2003, Gamble et al. 2008, Kraus 2009b). Human-mediated range expansions of geckos have mostly occurred through cargo shippments, allowing their successful establishment on distant islands (Garman 1892, Case 1975, Olmedo and Cayot 1994, Lever 2003, Gamble et al. 2008, Parent et al. 2008, Kraus 2009b, Hoskin 2011, Sturaro and Avila-Pires 2013, Silva-Rocha et al. 2019). Most research regarding invasive geckos has focused on nocturnal species of the family Gekkonidae due to their extensive presence throughout island ecosystems and their adverse effects on native fauna, for example, the common house gecko *Hemidactylusfrenatus* Duméril & Bibron, 1836 (Lever 2003, Jesus et al. 2005, Gamble et al. 2008, Kraus 2009b, Hoskin 2011, Torres-Carvajal 2015). Although several species of the diurnal family Sphaerodactylidae have also established non-native populations, they have been less studied, possibly due to their comparatively restricted geographical expansions, smaller body size and less evident ecological impact (Lever 2003, Kraus 2009b).

*Gonatodes* is a genus of small neotropical sphaerodactylid geckos with strong sexual dimorphism (Vanzolini 1968, Gamble et al. 2007). At least four out of the 33 species of *Gonatodes* are known to have successfully established populations outside of their native range: *G.albogularis* (*Duméril and Bibron 1836*), *G.antillensis* (Lidth de Jeude 1887), *G.caudiscutatus* (Günther 1859) and *G.vittatus* (Lichtenstein 1856) (*Kraus 2009b*). *Gonatodescaudiscutatus* is native to the Pacific lowlands and western foothills of the Andes in Ecuador and northern Peru, between 0 and 1800 m elevation (*Peters and Donoso-Barros 1970, Carvajal-Campos and Torres-Carvajal 2012, Sturaro and Avila-Pires 2013*). Non-native populations of *G.caudiscutatus* have established in the Galapagos Archipelago and on the eastern Andean slopes and Amazonian foothills of Ecuador due to human-mediated transoceanic and transmountain extra-range dispersions (*Vanzolini 1965, Wright 1983, Hoogmoed 1989, Olmedo and Cayot 1994, Carvajal-Campos and Torres-Carvajal 2012, Sturaro and Avila-Pires 2013, Cisneros-Heredia 2018*). *Gonatodescaudiscutatus* probably reached Galapagos as cargo stowaway on ships departing from ports along coastal Ecuador over the last two centuries and was first reported in San Cristóbal Island in 1891 (Olmedo and Cayot 1994, Cisneros-Heredia 2018). Nowadays, the species has established large populations in the highlands of San Cristóbal Island and smaller populations in the urban lowlands of San Cristóbal, ocurrences have been reported in Isabela, Baltra and Santa Cruz islands with no evidence of a stablished populations, but it is worth noting that this might be due to a lack of sampling efforts in these islands (Garman 1892, Wood 1939, Mertens 1963, Wright 1983, Hoogmoed 1989, Olmedo and Cayot 1994, Lundh 1998, Jiménez-Uzcátegui et al. 2007, Cisneros-Heredia 2018, Oleas Paz and Cisneros-Heredia 2019, Ramos Rojas and Cisneros-Heredia 2019, Ramos Rojas et al. 2020, Martínez Gómez and Cisneros-Heredia 2021).

There are no studies about the colonisation process of *G.caudiscutatus* in the Galapagos Islands and, in general, little is known about the species beyond its general morphology and distribution (Vanzolini 1968, Sturaro and Avila-Pires 2013). In contrast, the colonisation processes of the nocturnal, invasive gecko *H.frenatus* in Galapagos have been traced using molecular data, providing evidence of probable colonisation routes and points of origin (Torres-Carvajal 2015, Martins et al. 2022). These studies have contributed to a better understanding of the colonisation routes of small terrestrial vertebrates reaching islands as stowaways, a mechanism that has become more common over the last decade in Galapagos (Cisneros-Heredia 2018).

Across the globe, most species of introduced herpetofauna are understudied (Kraus 2009a) and genetic data for such species in the Galapagos are limited (Torres and Mena 2018). To develop better management and control tools for conservation in the Islands, it is essential to determine the most probable invasion routes, which requires knowledge of the genetic composition of the introduced species and their connections with the source populations (Chaves 2018). This study provides the first molecular insights into the genetic diversity of *G.caudiscutatus* along its native range in western Ecuador and about its introduction to the Galapagos Islands.

## Materials and methods

### Study area

We surveyed 34 localities across the native range of *G.caudiscutatus* in mainland Ecuador and 12 localities in San Cristóbal Island, Galapagos Archipelago (Suppl. material 1). Site selection was based on the known distribution of *G.caudiscutatus (Wright 1983, Hoogmoed 1989, Olmedo and Cayot 1994, Carvajal-Campos and Torres-Carvajal 2012, Sturaro and Avila-Pires 2013, Torres-Carvajal et al. 2016, Cisneros-Heredia 2018, Pazmiño-Otamendi and Carvajal-Campos 2019*) and previous fieldwork conducted by the Laboratory of Terrestrial Zoology of Universidad San Francisco de Quito USFQ between 2008 and 2018. Localities in western mainland Ecuador covered all known ecosystems where *G.caudiscutatus* has been reported in six Provinces (Esmeraldas, Bolívar, Manabí, Santo Domingo, Guayas, Los Ríos), including urban/periurban green areas in dry and humid lowlands and forested areas in dry, mesic and humid highlands. Localities in San Cristóbal Island covered ecosystems preferred by non-native populations of *G.caudiscutatus*, including urban/periurban green areas in the dry lowlands and agricultural and forested areas in the humid highlands.

### Data collection

Fieldwork was carried out in June–July 2019 in Galapagos and August- September 2019 in mainland Ecuador. Two researchers exhaustively searched for geckos at each locality for approximately 3 hours during the daytime, carefully looking under rocks, logs, rubble and other debris. Geckos were captured by hand and euthanised with benzocaine. Tail muscle samples were preserved in 90% ethanol and stored at −20°C until used and analysed at the Plant Biotechnology Lab of Universidad San Francisco de Quito USFQ. Voucher specimens were fixed in formalin and preserved in ethanol 70%. Specimens are deposited in the Zoology Museum at Universidad San Francisco de Quito, Quito, Ecuador (ZSFQ) under collection codes specified in Suppl. material 6. Insular specimens are currently kept under study at the Laboratory of Terrestrial Zoology of Universidad San Francisco de Quito USFQ. Upon completion of studies, we will deposit the specimens in a biological collection in the Galapagos Islands, following requirements by the Directorate of the Galapagos National Park and the Ministry of Environment of Ecuador. In the case of 10 samples specified in Suppl. material 6, the specimens escaped after releasing their tails. We sampled 56 individuals of *G.caudiscutatus* across all surveyed localities (Suppl. material 2).

### DNA extraction and amplification

Cell lysis was performed following the protocol described by Carranza et al. (1999). Tail tissue (0.5 cm) was macerated with sterilised plastic pistils and then incubated in a proteinase K digestion solution. Genomic DNA was extracted using a standard phenol/chloroform protocol (Kant and Coleman 2012). DNA concentration and quality were assessed using a Nanodrop 1000 Spectrophotometer and visualised in a 1.5% agarose gel. Each DNA sample was diluted to obtain a final concentration of 20 ng/µl. PCR was used to amplify fragments of the mitochondrial 16S rRNA gene (417 bp) and two coding nuclear genes: oocyte-maturation factor MOS (Cmos) (415 bp) and recombination activating gene 2 (Rag2) (410 bp). PCR amplification protocols established by Lobos (2013) and Gamble et al. (2008) were used with modifications specified in Suppl. material 3. Sequencing was commercially performed by Macrogen Inc. (Seoul, Korea).

### Sequence analyses

Sequence cleaning and aligning were performed using Geneious Prime 2020.0.5 software under default parameters. In order to assess genetic differentiation and genealogical relationships amongst *G.caudiscutatus* populations from San Cristóbal Island and mainland Ecuador, we built two trees using the three concatenated genes. The first tree was built using the Bayesian Inference (BI9 model under the default parameters in the Geneious Tree Builder option using Geneious Prime 2020.0.5 software (Biomatters 2020). The second tree was built using the Maximum Likelihood (ML) model on the IQ-tree online server (Trifinopoulos et al. 2016). We used ModelFinder (Kalyaanamoorthy et al. 2017) with 1000 bootstraps to determine the best-fit model. However, as both trees proved to be uninformative, they were removed from the study. Relationships amongst 16S haplotypes were assessed by constructing a haplotype median-joining network in PopArt1.7 (Leigh and Bryant 2015). We characterised the genetic variability of *G.caudiscutatus* using insular and mainland populations as operational geographic units by calculating nucleotide diversity (Pi) and haplotype diversity (Hd) in DNASP v.6 settings using default parameters (Rozas et al. 2017). A map was built using ArcGIS Pro (ESRI 2016) to show the haplotype distribution.

## Data resources

The GenBank accession numbers for the new sequences are MZ434825-MZ434876 for 16S sequences and MZ594479-MZ594565 for Cmos and Rag2.

## Results

We found individuals in 32 out of the 46 sampled sites and obtained sequence data of Rag2 for 40 individuals, of Cmos for 47 individuals and of 16S for 52 individuals. 16S sequences varied considerably (overall mean genetic distance of 24.31), but there were few differences in Cmos (overall mean genetic distance of 0.41) and no variation in Rag2 (overall mean genetic distance of 0). The results of the overall mean genetic distance for each gene and the genetic pairwise distances between all individuals for each gene can be found in Suppl. material 4. The 52 sequences of the 16S region corresponded to 33 individuals from 14 localities throughout western Ecuador and 19 individuals from San Cristobal Island. Given that the nuclear markers were not informative, we only used 16S to assess haplotype diversity and genealogical relationships.

**Figure 1.**
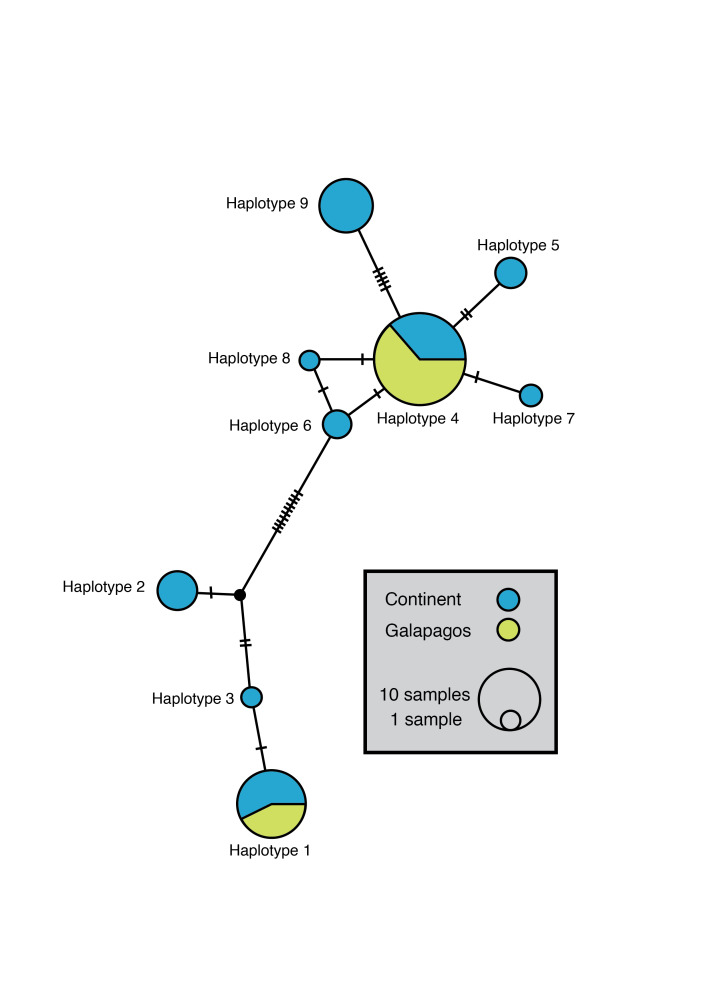
Haplotype network showing haplotypes found in mainland Ecuador (blue) and the Galapagos Island (green). Haplotype numbers correspond to those presented in Table 1 and Suppl. Material 5.

16S sequences showed 21 variable sites in the 403 base-pair region (Fig. 1). Nine haplotypes were found across all studied populations (Suppl. material 5) and the overall haplotype diversity was 0.7541. Mainland populations showed higher haplotype diversity (Hd) and nucleotide diversity (Pi) than San Cristobal populations (Mainland: Hd = 0.852 and Pi = 0.02135; San Cristobal Hd = 0.485 and Pi = 0.01516). Two major clusters separated by the highest number of mutations can be identified in the haplotype network (Fig. 1), each including haplotypes found both on the mainland and in the Galapagos.

**Figure 2.**
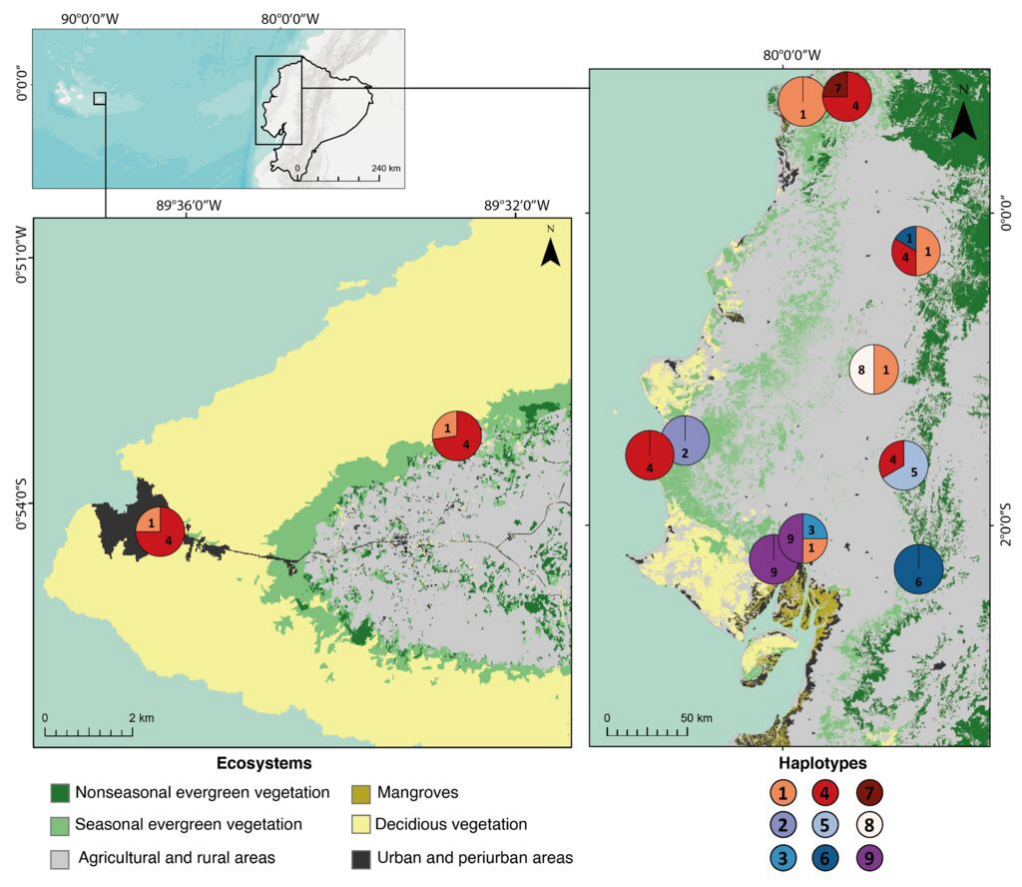
Genetic variation in surveyed populations of *G.caudiscutatus*. Pie chart colours correspond to haplotypes found in each locality. Numbers correspond to haplotypes detailed in Table 1. Pie charts represent the proportion of haplotypes found at each locality. Locality data are available in Suppl. material 2.

Haplotypes 1 and 4 were found widely throughout mainland Ecuador and are the only haplotypes found in the samples of San Cristóbal (Fig. 2, Table 1). Haplotypes 2 and 9 are more genetically distant from all other haplotypes by the number of mutations per site (*Fig. 1*, Suppl. material 5). These most diverging haplotypes are found in individuals from two populations in western Ecuador (Cerro Blanco and Agua Blanca), the only surveyed localities covered by old-growth seasonal deciduous lowland dry forests.

## Discussion

This study presents the first insights into the genetic diversity of native and introduced populations of *G caudiscutatus* in mainland and insular Ecuador. A total of nine haplotypes were found along the native range of *G.caudiscutatus* in western Ecuador. We found low nucleotide diversity values in mainland sites, with minor differences amongst populations. However, haplotype diversity was high; six haplotypes were unique to specific sampling locations, but their distribution did not correspond to any discernible biogeographic pattern. High haplotype diversity values have been reported for other *Gonatodes* that inhabit complex geographic ranges (Avila-Pires et al. 2012). The diverse orogenic and ecosystemic patterns of western Ecuador have been identified as an important factor driving genetic diversity in several clades (Willmott et al. 2001, Ron et al. 2006, Chaves et al. 2007, Waddell et al. 2018, Cisneros-Heredia et al. 2023). The unique haplotypes correspond to the only sampling localities in natural areas (Agua Blanca and Cerro Blanco), with all other haplotypes found in urban or periurban areas. As genetic, environmental and phenotypic variations are frequently geographically structured (Kaliontzopoulou et al. 2018), we suggest exploring how populations of those two localities differ in variables like morphology and behaviour from other populations of *G.caudiscutatus* across other natural and human-made habitats.

Two haplotypes were found on San Cristóbal Island, but none is exclusive to Galapagos and represent a small portion of the haplotype diversity from the mainland. Our analyses could not provide concrete evidence about the origin of the introduced populations of *G.caudiscutatus* in the Galapagos because there was no discernible genetic structure in the mainland: both island haplotypes were scattered throughout western Ecuador (Fig. 2). The presence of only two of the nine mainland haplotypes in the Islands could be attributed to a case of founder effect, where a small portion of the genetic pool of the mainland's population arrived to the Island with the colonising individuals, resulting in a less diverse genetic pool in insular populations. It is possible that, upon arrival, little genetic changes occurred, facilitated by the broad ecological tolerance the species showed on its native distribution and the similar environmental conditions shared between the native and introduced habitats, inhibiting further genetic diversification (Lee 2002). Furthermore, time since colonisation may be insufficient for genetic differences to accumulate in the specific markers under study (Chaves 2018).

Loss of genetic variation in introduced population due to bottlenecks during introductions has been reported to compromise the ability of populations to adapt to novel areas and limit their viability (Lee 2002, Kolbe et al. 2004). The low genetic diversity found in samples of *G.caudiscutatus* in the Galapagos may be related to its limited distribution in the Archipelago. The species has been present in the Galapagos Islands for about 130 years, but remains restricted to moist environments where it also shows considerable population fluctuations (Cisneros-Heredia 2018). Similarly, House Gecko, *Hemidactylusfrenatus*, arrived to Galapagos about 12 years ago and has haplotypes identical to those from its source populations (Melanesia) (Torres-Carvajal 2015); however, *H.frenatus* has become widespread and with increasing populations across most human-populated islands in Galapagos (Oleas Paz and Cisneros-Heredia 2019, Ramos Rojas et al. 2020, Martínez Gómez and Cisneros-Heredia 2021). Despite both species being in the order *Gekkota*, their invasion ecology shows significant differences that deserve further study.

Extra-range records of *G.caudiscutatus* are recurrently reported in mainland Ecuador and Galapagos, suggesting that the species may eventually hold a larger potential for colonisation of different lowland ecosystems (Sturaro and Avila-Pires 2013, Cisneros-Heredia 2018). This study is the first approach to describing the genetic diversity of insular and continental populations of *G.caudiscutatus*. Future studies with genomic tools could help to elucidate the dispersal history and genetic composition of *G.caudiscutatus* and other introduced geckos and small vertebrates in Galapagos and mainland Ecuador (Jesus et al. 2005, Rato et al. 2011, Avila-Pires et al. 2012, Pinto et al. 2018). This information could potentially help the development of strategies to prevent new colonisation events of geckos and other small terrestrial animals in the Archipelago, focusing on the points of origin and arrival and controlling colonisation routes (Allendorf et al. 2010, Chaves 2018, DeWoody et al. 2021).

## Supplementary Material

69DC94FF-6914-52CF-9E06-066CE76FDD30 10.3897/BDJ.11.e113396.suppl1 Supplementary material 1 Fieldwork locations Data type Localities information Brief description Nomenclature, province and coordinates for each continental locality visited during this research along with the date at which the fieldwork was conducted there. File: oo_904865.xlsx https://binary.pensoft.net/file/904865 Lía Altamirano-Ponce, Mateo Dávila-Játiva

E51E5621-6743-5D8F-B6AB-A4829E5044E5 10.3897/BDJ.11.e113396.suppl2 Supplementary material 2 Sample's information Data type Studied locations Brief description This document contains geographical information for the *Gonatodescaudiscutatus* tissue samples used on this study, including codes, waypoints, provinces on mainland Ecuador and the Galapagos Islands and sex of the specimen. File: oo_495667.xlsx https://binary.pensoft.net/file/495667 Lía Altamirano-Ponce and Mateo Dávila-Játiva

3CDE98CF-3290-5C0C-B0A9-10D7DA5F0C0D 10.3897/BDJ.11.e113396.suppl3 Supplementary material 3 Sequencing primers and PCR conditions Data type Primers and PCR conditions Brief description List of the sequencing primers and their respective PCR conditions (initial heating step, denaturation, annealing, extension and number of corresponding cycles) used for this work. File: oo_495688.xlsx https://binary.pensoft.net/file/495688 Lía Altamirano-Ponce

9A275F4E-7F69-5D97-9225-F20A304C9730 10.3897/BDJ.11.e113396.suppl4 Supplementary material 4 Genetic Pairwise and Overall Distances Data type Distance matrices Brief description Estimates of overall genetic diversity for each gene sequence (Cmos, Rag2 and 16S) and estimates of pairwise genetic diversity between all individuals for each gene (Cmos, Rag2 and 16S). File: oo_921131.xls https://binary.pensoft.net/file/921131 Gabriela Pozo, María José Pozo, Martín Terán-Velástegui, Maria de Lourdes Torres

7BDCA2F4-A73B-5DD7-A3DF-B413763DFD7B 10.3897/BDJ.11.e113396.suppl5 Supplementary material 5 Haplotypes information Data type Occurrences Brief description A detail of the sequences obtained for the 16S that conform to each of the nine haplotypes we found. File: oo_564990.xlsx https://binary.pensoft.net/file/564990 Lía Altamirano-Ponce

B7CE1D0C-1B38-5734-93EF-3761412E4123 10.3897/BDJ.11.e113396.suppl6 Supplementary material 6 Field and Collection codes Data type List of codes Brief description This is a list of the collection codes of the specimens used for this research project. File: oo_922246.xlsx https://binary.pensoft.net/file/922246 Mateo Dávila

## Figures and Tables

**Table 1.** Abundance and geographical distribution of haplotypes found in this study. Locality data are available in Suppl. material 1.

		**Haplotypes**								
Province	Locality	**1**	**2**	**3**	**4**	**5**	**6**	**7**	**8**	**9**
Esmeraldas	Acantilado	2								
	Esmeraldas City				3			1		
Bolivar	Caluma				1	2				
Guayas	Cerro Blanco									5
	Bucay						1			
	Pantanal Zoo	1		1						2
Manabí	Agua Blanca		4							
	Pto. Lopez				2					
Sto. Domingo	Santo Domingo City	3			2		1			
Los Ríos	Quevedo	1							1	
San Cristobal	Highlands	3			8					
San Cristobal	Lowlands	2			6					
Total		12	4	1	22	2	2	1	1	7
